# Active RFID Wake-Up Receiver Subsystem for Freight Wagon Localization Devices

**DOI:** 10.3390/s25041124

**Published:** 2025-02-13

**Authors:** Łukasz Krzak, Cezary Worek

**Affiliations:** Institute of Electronics, Faculty of Computer Science, Electronics and Telecommunications, AGH University of Krakow, 30-059 Kraków, Poland; worek@agh.edu.pl

**Keywords:** active RFID, wake-up receiver, WuRx, localization device, freight wagon positioning

## Abstract

This paper presents the concept, design, and performance analysis of an active radio wake-up and radio identification subsystem as part of an advanced localization device intended to operate within a large-scale freight wagon localization system. The system provides an efficient and cost-effective way to localize freight carriages, which, in the majority of cases, are currently not tracked. The localization device is battery-powered and uses an ultra-low-power radio interface for detecting wake-on-radio signals from nearby operator devices. The same interface is also used for communication within an ad-hoc wireless mesh network, which allows the localization devices to select the best device to send out localization information from the whole cluster through a cellular connection in order to minimize overall battery energy usage. The article presents the overall system architecture construction of the radio interface, including the wake-up subsystem, as well as the results of performance measurements.

## 1. Introduction

Railroad transportation is one of the most effective and environmentally friendly ways of transporting goods over long distances. According to the European Environment Agency, $CO_{2}$ emissions from rail transport are 3.5 times lower per tonne-kilometer than those from road transport [1]. However, the performance of rail freight transport in the EU remains unsatisfactory [2,3]. Among the many micro- and macroeconomic factors that affect this condition, one that is often described as challenging is the poor traffic management and suboptimal utilization of the current rail network and its assets [2]. One of the tools that helps improve logistic processes in this context is the automated and possibly real-time geolocalization of train carriages. However, in the case of freight wagons, such a system faces many technical challenges:

- It must operate at least over a continent, covering all possible train routes, coping with national requirements concerning radio spectrum usage;
- The localization devices must be mounted on each wagon individually, as the wagons are often swapped and mixed;
- The localization system should not rely on locomotive on-board units (LOBUs), as it complicates the adoption and deployment (more parties must be involved);
- Each localization device must be battery powered and the battery must last for at least 2 years (typical maintenance periods), as the freight wagons typically do not have access to a source of electric energy from the track [4].

As a result, many systems rely on specialized, battery-powered localization devices mounted directly on the wagons, utilizing satellite-based geolocation (GPS, Galileo, etc.) and cellular connection to send this information to the IT infrastructure. Some examples of such systems are SmartCargo from A1 Digital, deployed in Austria, and UDIV, deployed by the OLTIS Group in the Czech Republic. Much research and development has been conducted to build upon that architecture [5] or extend it [6].

Although using a cellular connection to upload wagon position to the cloud infrastructure has many benefits, such as worldwide coverage, high availability, and interoperability, it has one serious downside, which is relatively high energy usage. Based on an extensive one-year field study of such a prototype localization system, conducted by a major manufacturer of radiocommunication devices and systems for railways in Poland, Radionika, at least half of the energy available in the device was consumed by cellular connectivity. The main source of such high energy consumption is the long time (up to several minutes) required to establish the network connection. Data transmission uses a mature GPRS service, as the locations in which the train carriages operate are highly remote and at large distances from well-connected urban areas. The localization devices need to report their position at least once every 6 h and also when starting to move after a long stop. As a result, the batteries required for 2 year of operation become relatively large (two LSH 20 13Ah Li-ion batteries in the case of the above-mentioned prototype). This increases the cost of the device, makes the protective measures and maintenance harder, and significantly limits the miniaturization possibilities. Although some systems tried to use alternative uplink connection methods such as LoRaWAN [7] or are ready for modern long-range GSM services such as NB-IoT, the implementation of the required infrastructure in remote areas is progressing very slowly and does not yet provide adequate support for this kind of application.

Another requirement for the successful deployment of a freight wagon monitoring system, which is often overlooked, is the ability to easily identify and manage vehicles in the field. This requires the localization devices to be equipped with some sort of local communication interface that would allow the staff to interact with the device, which, in normal conditions, is mostly in deep energetic sleep mode, and thus is unable to receive any external signals. This brings even more benefits if the localization device is also combined with onboard sensors, which we will discuss in more detail in the next chapter.

The above-mentioned needs and challenges led to the collaboration between AGH University of Krakow and the Radionika company within an EU-cofounded project entitled “Research and deployment of a digital radio communication platform in railroad transportation”. One of the key goals of the project was to design a localization system for railway rolling stock that could provide near real-time information about the current position and use of the wagons while addressing known challenges and limitations.

The developed solution is based on a standard approach in the following way:

- It uses battery-powered positioning devices mounted on each wagon;
- Each device uses satellite signals for geolocation and precise clock synchronization;
- Each device is equipped with a cellular module to communicate with the infrastructure.

However, in order to significantly improve energy efficiency, an additional low-power radio communication module was introduced, along with a mesh-based collaborative network protocol, allowing organizing the nearby devices into clusters and electing a single device from each cluster (based on a specific energy consumption metric) to send positioning information on behalf of all devices in the cluster. While it allowed reducing the number of cellular connections from each node almost 10 times, it also opened a way to solve the second problem, which is the lack of a method to establish local communication with the positioning device, that would be active all the time without consuming significant energy. Unfortunately, the minimum power required to run radio reception in standard monolithic integrated radio transceivers, operating in sub-GHz bands is in the order of tens of milliwatts (for example, the CC1125 (Texas Instruments, Dallas, TX, USA) radio transceiver used in the project requires 1–5 mA at 3.3 V to run in so-called sniff mode [8]), but the acceptable energy budget for the local ad-hoc communication requires methods consuming 3 orders of magnitude less, in the order of tens of microwatts. This is why the device was equipped with a custom, ultra-low power wake-up receiver, designed specifically for the stated application.

The rest of the paper presents in detail the resulting wake-up receiver subsystem and is organized as follows. In Section 2, we describe the designed freight wagon localization system focusing on the above-mentioned improvements and introduce the wake-up radio subsystem. In Section 3, we present a state-of-the-art method concerning wake-up radio detectors. In Section 4, we discuss the construction aspects of the radio subsystem. In Section 5, we present the results of measurements and tests and give our conclusions in Section 6.

## 2. Overview of the Freight Wagon Localization System

**Figure 1:**
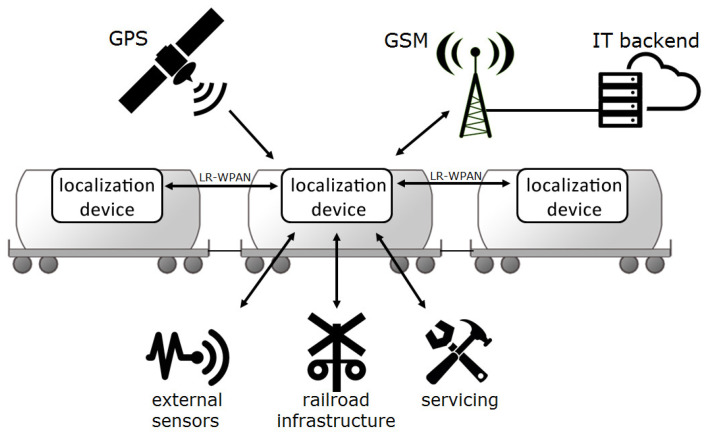
Illustration of the freight wagon localization system functionalities, featuring additional low rate wireless personal area network (LR-WPAN) radio interface.

The solution developed by Radionika and AGH requires that each monitored wagon be equipped with a battery-powered localization device that periodically wakes up, reads its GPS coordinates, and sends this information via a cellular network to the dedicated IT infrastructure for further processing. However, the project method introduced some major improvements over existing solutions, which are depicted in Figure 1 and which we will briefly describe in the following sections.

### 2.1. Construction of the Localization Device

**Figure 2:**
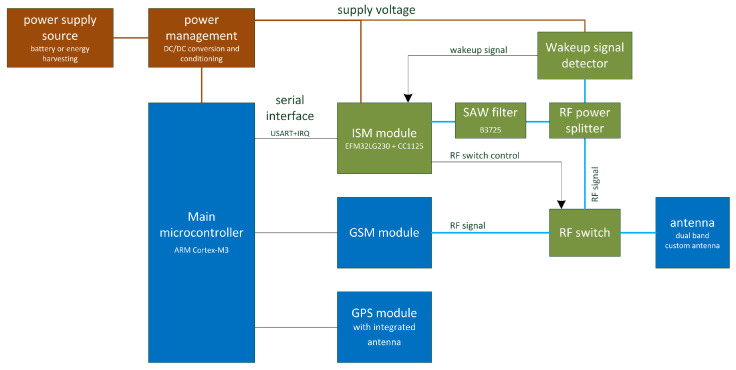
Block diagram of the localization device.

Figure 2 presents the block diagram of the localization device. The diagram was simplified in order to focus only on the components that are within the scope of this article. The components that constitute the wake-up radio subsystem and were designed specifically for this application are marked in green.

The localization device is powered with two LSH 20 13Ah Li-ion batteries (connected in series), but it could also be optionally powered by an energy harvesting module or a renewable energy source. The power management subsystem delivers operational voltages to all components. The system is controlled by an ARM Cortex-M3 main microcontroller. The platform integrates four different radio interfaces:

- GPS module with integrated antenna that receives satellite signal informing about location and time;
- GSM module that constitutes a long-range communication interface;
- Custom ISM (industrial, scientific, and medical band) module that delivers local communication capabilities between localization devices;
- Wake-up signal detector that enables peripheral sensors and other devices to asynchronously wake up the localization device and process external events.

**Figure 3:**
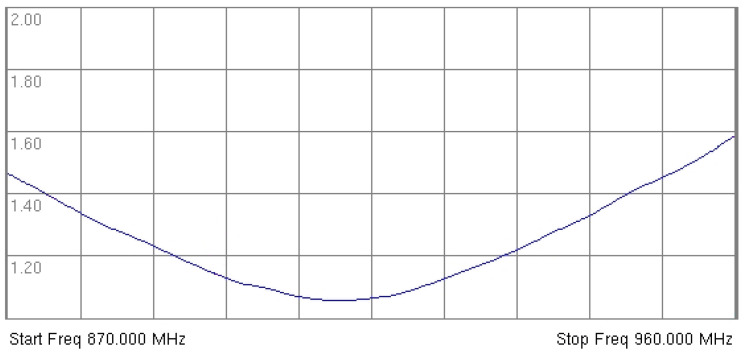
VSWR plot of the custom antenna designed for the localization device.

It is worth underlining that the GSM and ISM modules, as well as the wake-up signal detector, are utilizing the same antenna circuit, which is a custom multi-band antenna. The antenna was designed to cover both the unlicensed 863–870 MHz frequency bands as well as the 900 MHz and 1800 MHz GSM frequency bands available in Europe. Figure 3 presents the VSWR of the antenna in the low bands.

**Figure 4:**
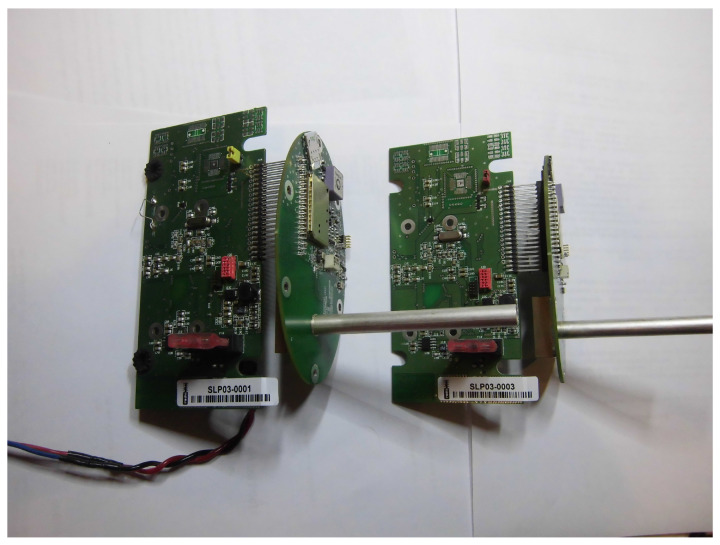
Pictures of the electronic part of the localization devices. The rounded top-mounted PCB includes the whole RF subsystem, including a custom antenna.

**Figure 5:**
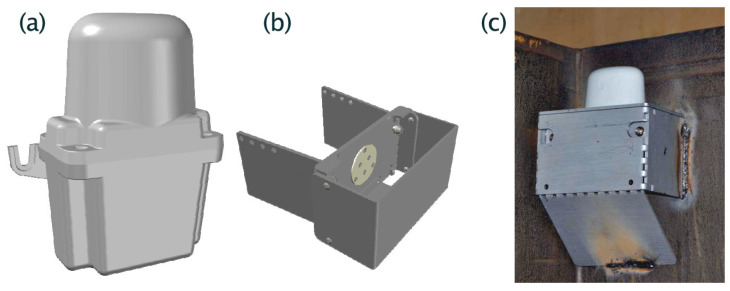
Pictures of the localization devices: (**a**) 3D model of the enclosure that holds the electronics, battery, and antenna, (**b**) 3D model of the steel pocket that is welded to the wagon side, (**c**) view of the localization device being mounted on a coal transportation wagon.

Figure 4 and Figure 5 illustrate the construction of the localization device, including the enclosure and the default mounting method.

### 2.2. Functionality Improvements over Existing Designs

The introduction of an additional ISM radio interface coupled with a wake-up radio signal detector operating in an unlicensed 863–870 MHz band in the EU brought several new functionalities to the freight wagon localization system. Due to the fact that the train carriages usually operate in groups, adding a short-range wireless communication interface allows the establishment of a local, low-power ad-hoc communication network operating as an LR-WPAN (low-rate wireless personal area network). Such a network serves several purposes, which we will briefly discuss below.

#### 2.2.1. Energy Savings Through Mesh Networking, Clustering, and Data Aggregation

A specialized, synchronous wireless communication protocol was developed, allowing the grouping of multiple nodes into clusters, aggregating data from such a cluster, and sending it via a cellular connection from a single cluster head node. The protocol has several interesting features:

- It allows devices (a.k.a. network nodes) to establish communication every fixed period of time (1 to 6 h). The ad-hoc operation session starts at predefined times in all localization devices thanks to GPS-based clock synchronization.
- It is stateless, meaning that each communication session is established ad-hoc, with no knowledge about past communication events. This allows the protocol to be insensitive to topology changes between sessions.
- The protocol allows the nodes to discover network neighbors and form the network within a fixed time (in the order of minutes).
- The protocol uses slotted TDMA with a fixed slot duration of 50 ms.
- The over-the-air baud rate is 100 kbps and the modulation characteristics follow the IEEE 802.15.4 SUN PHY specification.

The protocol organizes device operation into four phases:

- In the synchronization phase, the devices wake up roughly 3 min before the actual networking operation and use GPS receivers to obtain their position and synchronize the internal real-time clock to a global UTC time with approximately 5 ms accuracy. Next, they go to sleep until the exact moment the next phase is scheduled to start.
- In the advertising phase, which begins at nearly exactly the same moment in all nodes, the devices advertise their existence to other nodes, including information about the available charge. The goal of this phase is for each node to gather initial information about as many of its direct neighbors as possible.
- During the next phase called the association phase, the nodes use the information gathered during the advertising phase to form logical links over a slotted TDMA schedule. The goal is to assign to each pair of nodes a pair of time slots for exclusive communication, thus establishing an effective slotted communication schedule.
- In the final phase, called the clustering phase, the nodes use the established schedules to spread information about nodes within the possible cluster within a mesh network of a limited fixed radius so that collectively they can select the best candidate for the cluster head. Next, they send the request to this candidate to become the head of the cluster. If they receive confirmation, the clustering process ends. The cluster head is responsible for sending the position information about all the nodes that elected it to the infrastructure using a cellular connection.

The cluster head selection is completed based on a specific metric calculated by each node and broadcast during the clustering phase. This metric is based on the available charge in relation to the number of days the device operates since the last maintenance (when the battery is always replaced). This metric represents the deviation between the projected rate of battery charge depletion over time and the actual charge usage, recorded by the device. It is important to note that due to the fact that devices are rolled out in batches over time and the wagons are often swapped and mixed, many times the train will consist of devices in various stages of their projected lifetime. It is then crucial not to rely on the available charge metric, as the newest devices will always be depleted first. Instead, what we want to achieve is a uniform tempo of energy depletion, so devices with a slower charge depletion tempo are favored as cluster heads.

If, due to any circumstance, after the whole protocol operation is finished, the device fails to receive confirmation from the elected cluster head, it falls back to the default operation and sends its position through the GSM module on its own. However, through a detailed probabilistic modeling and behavioral simulation of the proposed protocol (out of the scope of this article), we have proven that this situation is very rare and the number of cellular transmissions from the group of nodes is vastly reduced. In the reference topologies studied, the charge required to relay the information about a wagon to the IT infrastructure, through the usage of clustering, is approximately 10 times smaller compared to the charge required for each node to send this information on its own, which is a significant energy savings [9].

#### 2.2.2. Communication with On-Board Sensors

The presence of a short-range radio communication interface with a wake-up radio signal detector allows us to introduce external sensors mounted on the wagon that make it possible to sense the state of the carriage, as well as the load storage conditions, and send this information along with the localization data. These sensors can be powered from a battery [10] or even through power harvesting (e.g., using vibration energy) [11,12].

Within the described system, the sensors use asynchronous communication with the localization device. Whenever a sensor has data to send, it transmits a wake-up radio signal to activate the localization device, then sends the data packet and receives an acknowledgment. Failure to receive the acknowledgment forces the sensor to repeat the process up to two more times.

#### 2.2.3. Communication with Railroad Infrastructure

Another function provided is the ability for the railroad infrastructure to request the localization device to report its position at the exact moment of passing a certain location, typically a mast with a stationary access point. This is useful from a logistics point of view whenever the arrival of a certain wagon at a predefined location must be recorded and time-stamped [13]. This operation of the access point is similar to a typical RFID interrogator [14]—it continuously broadcasts the wake-up radio signal (compliant with local duty cycle limitations) followed by an identification request and a listening period, in which the wagon localization device should respond. The response includes information about the wagon, including optional sensor data. The access point can now forward this information to the IT infrastructure using any wideband connection, for example, a cellular network.

#### 2.2.4. Support for In-Field Service Operations

Due to reliability constraints and rigid enclosure, the localization device does not have a user interface (buttons, etc.) and is usually mounted in difficult-to-reach places on the train carriage (see Figure 5). As the localization devices are mostly in power-down mode and operate according to their own internal schedule, in order to connect to them on demand, e.g., for servicing purposes, the wake-up radio signal is used. The wake-up radio signal transmitter (including the radio module) is coupled with a mobile terminal. The service staff employee can trigger the transmission of the wake-up signal, which in turn wakes up the localization device mounted on a nearby wagon. The adjustment of output power and the design of the directional antenna can be used to limit the number of devices waking up in a train station environment, where many wagons are in close proximity. The session initiated through the service staff proceeds in almost the same way as the session with the access point described above.

**Figure 6:**
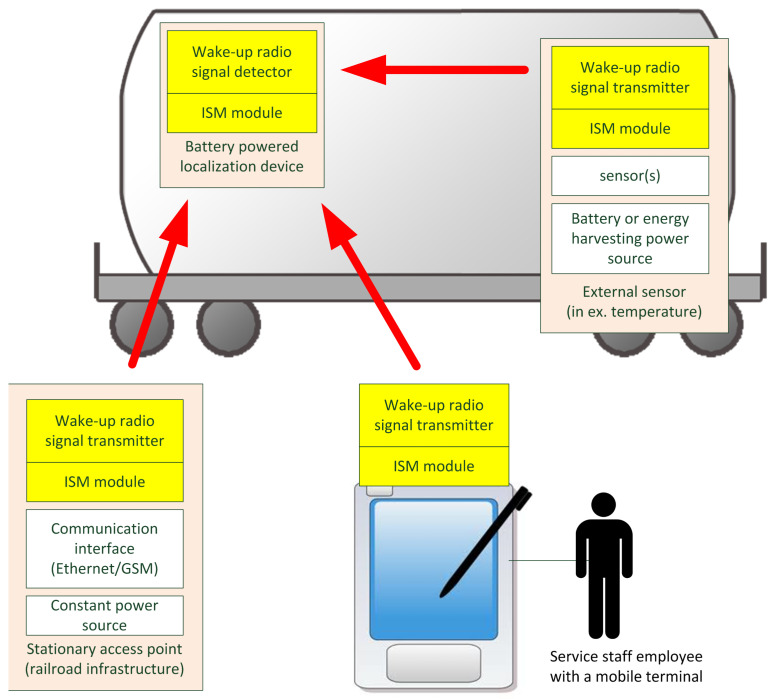
Illustration of the RFID features that are based on the wake-up radio subsystem. The red arrows show the wake-up signal path from various external devices, such as sensors on board of the wagon, stationary access points and handheld mobile terminals to the localization device.

Figure 6 illustrates all RFID features that are based on the wake-up radio subsystem.

## 3. Related Work

Wake-up radio (WuR) systems enable asynchronous wake-up of electronic devices, which are most often various types of autonomous sensors and nodes of wireless sensor networks (WSNs). Many WuR systems are based on an ultra-low power auxiliary radio path, most often called wake-up receivers (WuRx) [15,16,17,18]. Such receivers continuously monitor the RF spectrum for pre-specified event signatures (that is, wake-up messages) that tell the WuRx to enable the device to take further action, such as turning on the main radio or enabling a backscatter modulator [19]. In certain cases, WuRx systems allow for reducing the average energy consumption by continuous operation with low power consumption and activating the main broadband radio systems only after the occurrence/detection of an event and after transmitting the packets, switching back to the low-energy standby state.

Due to their practical application possibilities, they have been continuously developed for several decades, and the development of the Internet of Things (IoT) and Industrial IoT (IIoT) is currently expanding the areas of application [17,20,21]. The system solutions used in these designs are practically the same as in passive or semi-active RFID transponders; therefore, due to the diversity of applications, there are extensive articles in the literature presenting the current state of development of WuRx systems [15,17,20,22]. The practical dimension of the WuRx system solutions presented in specific applications, along with the description of the state-of-the-art method, can also be found in numerous doctoral theses [9,23,24,25].

In order to compare different WuRx solutions, many metrics need to be considered. The basic ones include the following [18,20]:

- Reading range: similar to radio systems, we can use the sensitivity metric, which is the easiest to compare. However, the effective communication range is greatly influenced by the selected operating band—most often used are unlicensed ISM bands: 125 kHz, 13.56 MHz, 433 MHz, 868/915 MHz, 2.4 GHz, and 5.8 GHz. Similar to RFID systems, the largest ranges are obtained in the 868/915 MHz band due to propagation conditions and legally permitted transmitter powers for the wake-up systems. For this reason, if we care about the reading range, comparisons should concern the same operating band and, additionally, the antenna system.
- Power Consumption: Since the WuRx system will be operating in standby mode all the time, it will often be a significant factor in the overall energy balance and, therefore, will heavily affect the battery lifetime of a device.
- Wake-up latency: The WuRx should respond to wake-ups with a reasonable delay, determined by the application requirements. In some applications, delays of a few seconds are acceptable, while in others, delays of microseconds are required. When designing the WuRx, there are ways to shape this parameter, but the general relationship is that the more stringent the latency requirements, the more power the WuRx receiver will draw.
- Immunity to interference and false alarms: WuRx systems may operate in an electromagnetic environment with high levels of interference, including many other radio emissions, and should, therefore, offer the ability to filter radio interference at an appropriate level.

In practical applications, it is also necessary to meet additional requirements related to the following:

- Resistance to environmental conditions: WuRx devices must operate reliably over a wide range of temperatures, humidity, and supply voltages, as well as in the presence of water, dust, and vibrations, and should not require any special calibration.
- Dimensions and weight: Minimizing these parameters in WuRx systems is strongly related to the economics and size of the antenna system and is especially important for small IoT/IIoT devices that rely on small batteries or energy harvesters.
- Economics: related to the time and costs of the project, the costs of starting production, the production itself, the availability of components, etc. In the simplest example, we can compare better technical parameters but more expensive solutions in the form of a specialized ASIC integrated circuit to solutions based on discrete elements [22].
- Compliance with standards: In order to introduce a product to the market, it is necessary to meet the legal requirements stated in relevant standards concerning electromagnetic compatibility, radio spectrum usage, environmental hazards, etc.

## 4. Construction of the Wake-Up Radio Subsystem

### 4.1. Selection of Operating Frequencies

**Figure 7:**
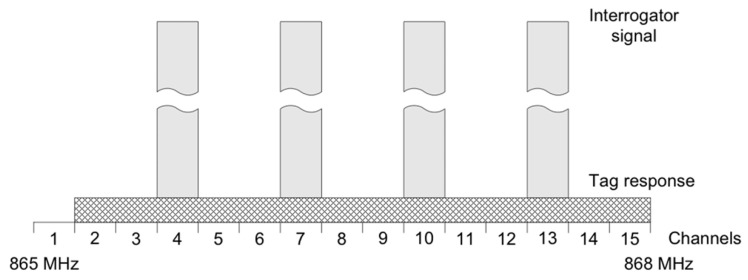
Channel plan for 865–868 MHz frequency band, according to ETSI EN 302 208 V3.3.1:2020.

Due to the principle of operation, where the wake-up radio signal activates the otherwise disabled localization device, causing it to broadcast a radio signal, the system needs to be treated as an RFID (radio frequency identification) system. Within EU countries, this poses restrictions and requirements that are stated in the ETSI 302 208 standard [26]. The standard divides the applicable 865–868 MHz frequency band into 15 channels, each 200 kHz wide. Channels 4, 7, 10, and 13 are high-power channels designated for RFID interrogators to broadcast the interrogator signal—see Figure 7. In these channels, up to +33 dBm (2W), effective radiated power is allowed for a maximum continuous time of 4 s, after which a minimum quiet period of 100 ms is required.

In order to maximize the frequency separation between the wake-up signal and the communication signal, channel 4 was chosen. The wake-up signal is broadcast with a center frequency of 865.7 MHz, and the center frequency for the communication for the ISM module was selected to be 869.9 MHz.

### 4.2. Description of the Electronic Construction

**Figure 8:**
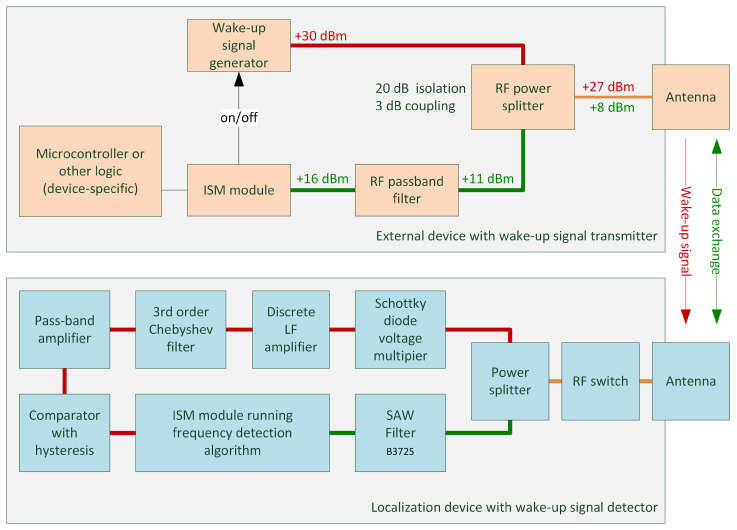
Illustration of the wake-up subsystem architecture. The top part presents an external device with a wake-up signal transmitter, capable of generating the wake-up signal (red and orange signal path) as well as transmitting and receiving data (green and orange signal path) once the localization device is woken up and the session is established. Red power values indicate power levels of the wake-up signal and green power values indicate power levels of the RF signal produced by the ISM radio module. An important aspect of the design is the RF power splitter, which has 20 dB isolation so that the wake-up signal leaking into the ISM module is at an acceptable level. The lower part of the diagram presents the components responsible for processing the received wake-up signal (red and orange signal path) and those that take part in regular data exchange (green and orange signal path) once the localization device is woken up and the session is established.

The wake-up radio feature uses an active radio approach, in which a dedicated ultra-low-power radio signal detector is always on, listening to the radio channel and searching for a signal with certain expected attributes [15,22]. The presented patented wake-up radio subsystem is based on a dedicated radio signal detector, capable of listening while consuming very low energy. The architecture of the wake-up subsystem is presented in Figure 8, and detailed schematics are available in Appendix A.

The wake-up signal transmitter is a device that integrates a wake-up signal generator along with a reused ISM module and optional device-specific control logic (microcontroller) and/or sensors. In order to wake up a nearby localization device, the ISM module enables the wake-up signal generator that generates a special high-frequency signal (marked in red in Figure 8). The system uses a wake-up signal at 865.7 MHz with AM amplitude modulation (sinusoidal modulating signal, 217 Hz modulation frequency, 80% modulation depth). The band was chosen because of the possibility of using a single antenna for the ISM radio module, the GSM module, and the wake-up detector. The power levels indicated in red show the maximum wake-up signal power coming from the generator at various stages of the RF signal chain. The wake-up signal is fed to the antenna through a power splitter, which allows the signal to be isolated from the second signal path, which leads to the ISM module through an additional RF passband filter, providing even more attenuation on the wake-up signal and other possible strong interference signals (GSM, GSM-R). The green color indicates the power levels of the RF signal produced by the ISM radio module. Both signal levels were designed to meet the requirements concerning radio signal broadcast regulations, assuming the use of an antenna with up to 6 dBi gain.

Once the wake-up signal reaches the localization device (see Figure 8), it is detected by the wake-up signal detector. The signal from the antenna passes through a non-powered RF switch SKY13377-313LF (Skyworks, Irvine, CA, USA), which was selected due to its low attenuation, only 7.3 dB, in this mode of operation. The signal is then split into the ISM module through an additional B3725 SAW filter (TDK Corporation, Tokyo, Japan) as well as the actual detector circuit. At the detector input, the voltage of the signal is multiplied using DC-biased Schottky diodes (approximately 1.5 µA). As an envelope detector, a three-stage Dickson voltage multiplier was used in which Schottky diodes were polarized with a DC-biased current (approximately 1.5 µA) to increase their sensitivity [27,28]. The demodulated signal is then amplified by an ultra-low-power and low-noise discrete amplifier with series feedback, based on the BC847B and BC857B (NXP Semiconductors, Eindhoven, The Netherlands) transistor pair. Only on discrete elements has it been possible to build an amplifier with a very low current consumption of 2.4 µA and a sufficiently large gain bandwidth over 100 kHz. Next, it is filtered using a third-order Chebyshev low-pass filter and amplified using a pass-band amplifier (10× amplification, Q-factor = 5) based on OPA2369 (Texas Instruments, Dallas, TX, USA) with a current consumption of about 1.5 µA. The resulting signal is fed through two RC filters with different constant times to a comparator with hysteresis, which worked as a data slicer with a current consumption of about 0.8 µA, and then fed to the dedicated wake-up interrupt input of the ISM module. In case there is no wake-up signal present, the ISM module is put into deep sleep mode. Once the signal activates the comparator, the firmware running in the ISM module starts to count the frequency of the received impulses, as well as the number of periods, which further qualifies the signal as a valid wake-up. In the presented solution, the number of required valid impulses was set to 22, which gives the wake-up latency of approximately 106 ms. In case the signal matches all criteria, the ISM module is woken up to an active state and can further participate in the communication with the wake-up signal transmitter using the main mode of communication. In other cases, the module is put back into a deep, energetic sleep.

**Figure 9:**
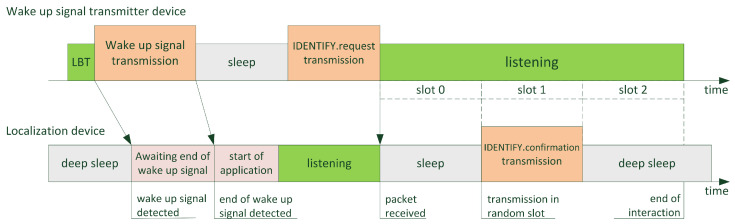
Illustration of the communication scheme between wake-up signal transmitter and woken up localization device. Green periods indicate state of radio listening, orange periods indicate state of radio transmission, grey periods indicate state of sleep and pink periods mark the activation of the wake-up signal detection algorithm.

According to legal requirements, before emitting RF power, the wake-up signal transmitter checks if the radio channel is free using a listen-before-talk (LBT) algorithm. If it is, it broadcasts the wake-up signal for approximately 220 ms. This is sufficient, as the overall wake-up detection latency does not exceed 106 ms. Next, the device switches to reception mode and, after approximately 90 ms, broadcasts an IDENTIFY.request packet. This packet carries information about the wake-up transmitter address, as well as the length and number of time slots that immediately follow this packet, in which the woken-up localization devices may broadcast their responses. The length and number of slots are selected according to the application requirements. Figure 9 illustrates this process.

When the wake-up signal is detected in the localization device, the algorithm waits for the end of the signal, and after some time needed to start the application, it switches the ISM module to reception mode. Once the IDENTIFY.request packet is received, the device chooses a random slot, in which it responds with the IDENTIFY.confirmation packet. This packet carries the address of the localization device. In this way, both devices exchange each other’s identification data. Optionally, the IDENTIFY.request packet can carry a list of explicit node addresses that should maintain communication during the slotted channel access period. In such a case, only listed devices respond with an IDENTIFY.confirmation packet, which is useful in the particular case of a service device that needs to wake up only a specific localization device. The slotted channel access may continue to exchange additional application-specific information such as sensor values, etc. At this stage of project development, it is assumed that if the localization device being awakened receives important information from the sensors, railway infrastructure, or servicing device it is rather urgent and should be delivered to the IT infrastructure as soon as possible. This is why the localization device uses the GSM service immediately after the described interaction, to contact the supervising system and notify about the encountered event. In the future, it will also be possible to aggregate such data in the localization device and exchange them during the clustering process.

## 5. Measurement Results

### 5.1. Current Consumption

In the case of no wake-up signal, the detector was measured to consume no more than 7 µA of power supply current (when powered from 3 V). The current consumed by the MCU operating in shut-down mode is 5 µA (same supply voltage). Thus, the current consumed by the complete radio module in power-down mode with the active wake-up detector is approximately 12 µA (36 µW).

### 5.2. Sensitivity

**Figure 10:**
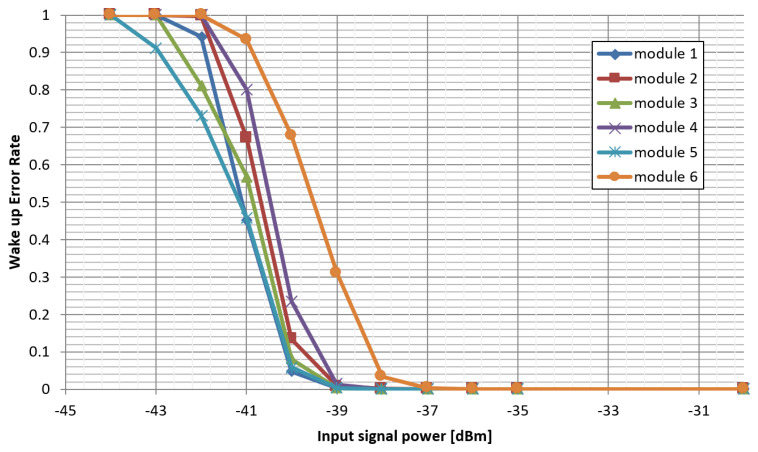
Results of sensitivity measurements.

For the purpose of measuring the sensitivity of the wake-up radio detector, a metric called the wake-up error rate (WER) was defined. Similarly to the packet error rate (PER), it is the ratio between failed wake-up radio transactions to the overall number of wake-up attempts. A failed wake-up transaction is the event in which the wake-up detector failed to recognize the wake-up signal, despite it being broadcast by the transmitter. Figure 10 presents the results collected from six prototype modules tested. For clarity, the measured points were linearly interpolated. As expected, the resulting plot resembles a typical characteristic of PER.

The sensitivity of the WuRxs in this system has been evaluated to be −38 dBm, at which the ratio of reported missed or false wake-up events to the number of total wake-up events generated was below 1%. When evaluating this result against other wake-up radio solutions, we have to note that the effective sensitivity is reduced by the RF signal path that allows the use of a single antenna (see Figure 8), which attenuates the wake-up signal reaching the detector by 12–14 dB. When measured in a stand-alone configuration, WuRx has a sensitivity of −52 dBm. Taking into consideration that the solution consumes approximately 21 µW of power and is realized using discrete, common elements, the resulting sensitivity was considered satisfactory, especially since it shows high selectivity and resistance to interference signals.

### 5.3. Effective Range

In addition to the wake-up radio detector sensitivity measurements, a practical range test was also performed in three locations on the campus of AGH University in Krakow.

**Figure 11:**
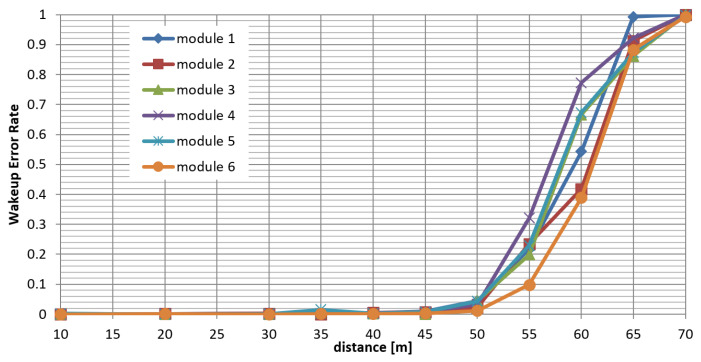
Results of range measurements.

The tests were carried out under line-of-sight conditions in an open field location. In the test, a wake-up signal transmitter was sending 10 sessions of 1000 signal bursts each to the module that was operating as a wake-up detector. The distance between the transmitter and the receiver was changed, and the wake-up error rate was noted in the receiver. Figure 11 presents the results of a range test performed independently for six modules with a wake-up signal transmitter power of +27 dBm, AM modulation frequency of 217 Hz, modulation depth m = 80%, transmitter antenna gain +6 dBi, and receiver antenna gain +2 dBi. The wake-up error rate was averaged. The measured points were linearly interpolated for clarity.

In both cases, the practical range of the wake-up subsystem was estimated as 40–45 m, at which the WER metric is close to 1%.

### 5.4. Selectivity

**Figure 12:**
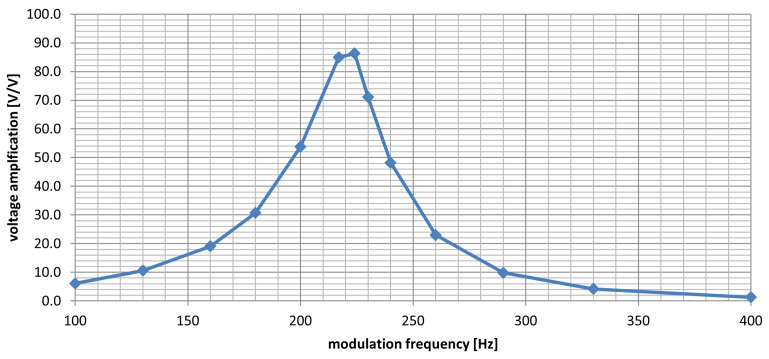
Results of selectivity measurements show the voltage amplification factor in the wake-up detector signal chain as a function of modulation frequency.

In order to determine the frequency selectivity of the WuRx, a signal from an RF generator was fed to its input, with a level of −38 dBm. The signal was AM amplitude modulated, with a modulation depth of 80%. The modulating frequency was changed between 100 and 400 Hz, and the output signal was measured at the input of the U7A op-amp pin 1 (see Appendix A). This way, the signal path also included the diode detector. The results of the voltage amplification as a function of modulation frequency are shown in Figure 12.

The presented frequency response was designed to provide strong rejection to interference signals that could falsely trigger the wake-up radio event. Frequencies above 300 Hz are heavily attenuated. The second part of the signal qualification, which also strengthens the selectivity, is done by the frequency detection algorithm running in the ISM module, as described in Section 4.2.

It is also worth noting that while the measurements presented in Figure 12 were taken at relatively low input power, the circuit shows good selectivity at input signal levels between −50 dBm and up to −20 dBm at which the wake-up detection happens only for modulation frequencies between 205 and 225 Hz. What is more, the circuit is not blocked with signals even up to 0 dBm input signal power, which is important in cases when auxiliary devices with wake-up signal transmitters are operating close to the positioning device.

## 6. Conclusions

This article presents a practical implementation of the WuRx low-power wake-up radio system that was successfully used in a railway wagon location system, and the presented solution architecture was unique enough to gain patent protection in Poland. It should be emphasized that the presented solution met all the requirements related to the operation of a radio device on the railway, i.e., the requirements of radio and railway standards, and functional tests were extended, among others, to include range tests in open space, where a distance of 45 m was achieved at 1% WER. The distinguishing feature of the presented solution is the operation of three independent radio systems (GSM, LR-WPAN network, WuRx) with a single antenna and an RF switch that is not powered and does not consume any current. However, this reduced the effective sensitivity of the WuRx system to a level of −38 dBm, even though it independently achieved a sensitivity of −52 dBm. The proposed design is based on the classic architecture with a voltage multiplier with pre-polarized Schottky diodes, and the low-frequency amplifier and filters were built from discrete elements. Due to the wake-up modulation frequency in the range of about 217 Hz and applied filtration, the selectivity of the wake-up system was ensured, and the wake-up latency parameter did not exceed 106 ms. The use of a sinusoidal modulation signal in the wake-up system transmitter caused the output signal to be spectrally narrow and pure, which in turn made the detection algorithms perform better. All tests performed confirmed the fully correct operation of the designed WuRx system, which did not require any tuning and added several improvements to the functionality of the entire freight wagon positioning system.

## 7. Patents

The localization device solution gained patent protection in Poland as “Method for radio communication and activation of a position locators and the position locator”, Application number P.412566, Registration number Pat.228002, application date: 29 May 2015, registration date: 14 September 2017. Inventors: Cezary Worek and Łukasz Krzak, Applicant/Holder: Radionika sp. z o.o., Kraków, Poland.

## Data Availability

Data are contained within the article.

## Appendix A

Detailed schematics of the wake-up signal detector.
